# Draft genome assembly and transcriptome sequencing of the golden algae *Hydrurus foetidus *(Chrysophyceae)

**DOI:** 10.12688/f1000research.16734.3

**Published:** 2019-10-07

**Authors:** Jon Bråte, Janina Fuss, Shruti Mehrotra, Kjetill S. Jakobsen, Dag Klaveness

**Affiliations:** 1 Section for Genetics and Evolutionary Biology (EVOGENE), Department of Biosciences, University of Oslo, Oslo, 0316, Norway; 2 Institute of Clinical Molecular Biology, Christian-Albrechts-University Kiel, Kiel, 24118, Germany; 3 Section for Aquatic Biology and Toxicology (AQUA), Department of Biosciences, University of Oslo, Oslo, 0316, Norway; 4 Centre for Ecological and Evolutionary Synthesis (CEES), Department of Biosciences, University of Oslo, Oslo, 0316, Norway

**Keywords:** Hydrurus foetidus, Chrysophyceae, golden algae, genome, transcriptome, Nanopore, PacBio

## Abstract

*Hydrurus* *foetidus* is a freshwater chrysophyte alga. It thrives in cold rivers in polar and high alpine regions. It has several morphological traits reminiscent of single-celled eukaryotes, but can also form macroscopic thalli. Despite its ability to produce polyunsaturated fatty acids, its life under cold conditions and its variable morphology, very little is known about its genome and transcriptome. Here, we present an extensive set of next-generation sequencing data, including genomic short reads from Illumina sequencing and long reads from Nanopore sequencing, as well as full length cDNAs from PacBio IsoSeq sequencing and a small RNA dataset (smaller than 200 bp) sequenced with Illumina. The genome sequences were combined  to produce an assembly consisting of 5069 contigs, with a total assembly size of 171 Mb and a 77% BUSCO completeness. The new data generated here may contribute to a better understanding of the evolution and ecological roles of chrysophyte algae, as well as to resolve the branching patterns at a larger phylogenetic scale.

## Introduction

Here, we present extensive genome sequencing data, including a hybrid assembly, as well as transcriptome data of mRNAs and small RNAs of the golden algae *Hydrurus foetidus* (Villars) Trevisan.

There has been considerable interest in the golden algae for many reasons: they are ecologically diverse, important as primary producers (phototrophs) in oligotrophic to dystrophic lakes ( Kristiansen, 2005; Nicholls & Wujek, 2015), some are also mixotrophs, phagotrophs or osmotrophs ( Kristiansen & Preisig, 2001; Pringsheim, 1963). The chrysophytes span a large range of feeding and nutrient uptake modes ( Kristiansen, 2005) and therefore play a significant role in aquatic food webs. Chrysophytes also make up a significant fraction of sequence reads and novel operational taxonomic units in clone libraries from freshwater environmental samples ( del Campo & Massana, 2011).

However, chrysophytes have also attracted considerable interest from an evolutionary point of view. They belong to the division (phylum) Heterokonta ( Cavalier-Smith, 1986) (Stramenopila according to Adl *et al*., 2019), an immensely diverse group of eukaryotes with many basal branches in the phylogeny still not resolved, despite numerous molecular phylogenetic studies, including multigene phylogenomics (e.g. Burki, 2014; Grossmann *et al*., 2016; Riisberg *et al*., 2009; Scoble & Cavalier-Smith, 2014). One reason for this is the presence of cryptic species and many groups with extremely similar morphology ( Grossmann *et al*., 2016). Another reason is the complex evolutionary history of the Heterokonta, including an elaborate plastid evolution (e.g. Kim *et al*., 2019) and heterotrophic lineages which have lost the plastids altogether ( Graupner *et al*., 2018; Pringsheim, 1963). The lack of genomic or transcriptomic data from many taxa, and even whole orders, which limits the power of multigene phylogenies ( Beisser *et al*., 2017), is yet another motivation for genomic and transcriptomic studies. However, recently, there has been a significant addition of transcriptomic data for chrysophyte taxa (e.g. Beisser *et al*., 2017; Graupner *et al*., 2018; Keeling *et al*., 2014; Kraus *et al*., 2019; Lie *et al*., 2017).

*Hydrurus foetidus* is not a typical representative of the golden algae. It is macroscopic and benthic (e.g. Klaveness *et al*., 2011; Rostafinski, 1882: Tab II, Szklarczyk, 1953), whereas most chrysophytes are microscopic single cells or colonial plankton ( Sandgren, 1988; Kristiansen, 2005). Furthermore, *Hydrurus* is native to polar, peri-glacial and alpine rivers in Norway and similar regions around the world (e.g. Klaveness, 2019; Rott *et al*., 2006; Rott & Schneider, 2014) and can only live in cold waters (2–10°C) ( Bursa, 1934; Kann, 1978). Members of the *Hydrurus* clade may cause colored snow and ice, and may be found on permanent ice sheets ( Klaveness *et al*., 2011; Lutz *et al*., 2018; Remias *et al*., 2013).

*Hydrurus* has a number of peculiar morphological characteristics relevant for understanding chrysophyte and heterokont evolution. Although it is multicellular, the cells in the thalli are not physically connected, and under some growth conditions the cells may slide away from each other in their wall-less polysaccharide tubes, or be released as single-celled swarmers ( Klaveness *et al*., 2011). Other characteristic features, which may be considered primitive for a thallose alga, are contractive vacuoles, often more than one in each cell ( Fott, 1959; Klaveness, 2019).

We have assembled a draft genome of *Hydrurus foetidus* using a combination of short-read Illumina sequencing and long-read Nanopore sequencing. The assembly consists of 5069 contigs yielding a total size of 171 Mb and a 77% BUSCO completeness. In addition to the deep genomic sequencing, we have also sequenced full-length poly(A) transcripts using PacBio IsoSeq, as well as sequencing the expressed small RNAs. This extensive dataset will be important, not only for studies of heterokont and chrysophyte evolution but also for elucidating the genetic mechanisms behind cold water adaptation, like the production of polyunsaturated fatty acids ( Klaveness, 2017) and the regulation of a complex multicellular lifestyle.

## Materials and methods

### Culturing of *H. foetidus*

The specimen of *Hydrurus foetidus* (Villars) Trevisan (strain G070301) used in this study was sampled from the river at the Finse Alpine Research Center (60°36' N. 07°30' E) in March 2007 and is currently kept in culture at University of Oslo. The photosynthetic *H. foetidus* was cultured at 4°C with a 14:10 hour light/dark cycle and kept in an adapted Guillard & Lorensen’s WC (Wright’s Chu) medium ( Guillard & Lorenzen, 1972) as described by Klaveness & Lindstrøm (2011). To prepare for DNA isolation, the growth of large thalli was promoted by repeated transfer of individual thalli into fresh culture media. Large thalli (0.5–1.0 g wet frozen weight) were collected by removal from the culture medium and immediate transfer to -80°C and storage until further processing. The culture will be deposited in a special culture collection, at the Fraunhofer Culture Collection of Cryophilic Algae (CCCryo).

### Illumina sequencing of genomic DNA

Isolation of genomic DNA for Illumina sequencing was performed as part of an unpublished master project ( Mehrotra, 2018). Briefly, six individual thalli were used for the DNA isolation. DNA isolation was performed using the DNeasy Plant Mini Kit from Qiagen (Qiagen Inc., Valencia, CA, US). To ensure efficient lysis and homogenization of the external polysaccharide sheath, a few titanium beads were added to the frozen samples and the tubes were shaken using TissueLyser II machine (Qiagen Inc., Valencia, CA, US) for four minutes. After the addition of the lysis buffer and the RNase, tubes were placed in a thermomixer set at 65°C and 800 rpm with 20 second intervals for 30 mins. After adding Buffer AP2 to the lysate, the incubation was done on ice for 15 minutes to allow for better precipitation of the polysaccharides. Further, the extraction kit protocol was followed as is, until the second elution step. Here we reused the flow through from the previous step elution to avoid excessive dilution of the samples. Afterwards, the samples were de-salted and concentrated by ethanol precipitation and resuspension in 100 μl of Milli Q water. Finally, the samples were concentrated even further by pooling all the samples and freeze drying with a Leybold-Heraeus Lyovac GT2 (Leybold-Heraeus, Köln, Germany). The final sample had a concentration of 104 ng/μl and 260/280 ratio of 1.85 and 260/230 ratio of 1.47 as measured on a Nanodrop ND-1000 (ThermoFisher, MA, US).

The isolated and freeze-dried genomic DNA was sent to the Norwegian Sequencing Center (NSC) at the University of Oslo for library preparation and sequencing. Briefly, the sequencing library was made with the Illumina Truseq LT DNA kit (following the rapid mode protocol), with 600–700 bp fragment size and sequenced on two lanes of Illumina HiSeq 2500 with 250 bp paired-end reads ( Table 1).

**Table 1.** Overview of datasets produced in this study.

Dataset	Description	Accession
Hfoetidus_ACAGTG_L001_R1_001.fastq.gz Hfoetidus_ACAGTG_L001_R1_001.fastq.gz	Genomic DNA sequenced with Illumina HiSeq 2500.	ERR2882522
Hfoetidus_ACAGTG_L002_R1_001.fastq.gz Hfoetidus_ACAGTG_L002_R1_001.fastq.gz	Genomic DNA sequenced with Illumina HiSeq 2500.	ERR3188711
Hydrurus_nanopore_fastq_files.tar.gz	Basecalled Oxford Nanopore reads	ERR2887871
IsoSeq_1-2kb_polished_low_qv_consensus_isoforms.fastq.gz	mRNA sequenced with PacBio SMRT RSII.	ERR2882521
IsoSeq_1-2kb_polished_high_qv_consensus_isoforms.fastq.gz	mRNA sequenced with PacBio SMRT RSII.	ERR2869477
IsoSeq_2-3kb_polished_low_qv_consensus_isoforms.fastq.gz	mRNA sequenced with PacBio SMRT RSII.	ERR2869481
IsoSeq_2-3kb_polished_high_qv_consensus_isoforms.fastq.gz	mRNA sequenced with PacBio SMRT RSII.	ERR2869478
IsoSeq_3-6kb_polished_low_qv_consensus_isoforms.fastq.gz	mRNA sequenced with PacBio SMRT RSII.	ERR2869484
IsoSeq_3-6kb_polished_high_qv_consensus_isoforms.fastq.gz	mRNA sequenced with PacBio SMRT RSII.	ERR2869483
1-Hfo-miRNA_S6_R1_001.fastq.gz	Small RNA sequenced with Illumina NextSeq 500.	ERR2869485
pilon_round3.fasta.gz	Draft genome assembly	GCA_900617105.1

### Nanopore sequencing of genomic DNA

Genomic DNA was isolated from two thalli as described above, except that tissue lysis was done using MagNA Lyser Green Beads (Roche, Penzberg, Germany) and shaken for 15 sec at 4 m/sec and the incubation at 65°C was done for 10 min. In addition, the supernatant (after adding buffer AP2) was run through QiaShredder columns to further homogenize the lysate. The DNA was eluted (twice, but re-using the elution buffer) in 50 µl AE elution buffer. To further clean and concentrate the samples, the samples were pooled and cleaned using the Zymo DNA Clean & Concentrate kit (Zymo Research, CA, US). The sample was double-eluted (as before) in 50 µl kit provided elution buffer (DNA concentration 61.8 ng/μl, 260/280 ratio of 1.93 and 260/230 ratio 2.05 as measured on a Nanodrop).

DNA sequencing was done using the MinION (MIN-101B) sequencer, the R9.5 Flow Cell and following the SQK-LSK108 protocol (version GDE_9002_v108_revT_18Oct2016) (Oxford Nanopore, Oxford, UK). Approximately 1 µg of starting DNA was used and inspection of the DNA on a 0.7% agarose gel run at 30 volts from 18 hours showed that the majority of the DNA was between 20-30 kbp, but with a long tail of shorter fragments. The sequencing was run using the MinKNOW software (Oxford Nanopore, Oxford, UK; downloaded October 2017) on an iMac and stopped after 36 hours. Base-calling of the raw Nanopore sequence data was done using Albacore v.2.1.10 (Linux, Python 3.5 version; Oxford Nanopore, Oxford, UK) with default settings. The process was run on the Abel computing cluster at the University of Oslo.

### PacBio transcriptome sequencing

Total RNA was isolated from one frozen thallus using Qiagen RNeasy Plant kit, including a QiaShredder column and lysis using MagNA lyser beads as described above, otherwise following the kit protocol. Isolated RNA was sent to NSC for library preparation and PacBio sequencing. Three size fractions (1-2 kbp, 2-3 kbp and 3-5 kbp) were prepared using the IsoSeq library preparation protocol (with selection of polyadenylated transcripts) and sequenced on RSII SMRT cells (Pacific Biosciences, CA, US) ( Table 1 and Table 2).

**Table 2.** Summary of the read numbers in the different file types of the IsoSeq data set.

	Size fraction						
Library	< 1kb	1-2kb	2-3kb	3-4kb	4-5kb	5-6kb	> 6kb
1-2kb_high	7310	31953	17	0	0	0	0
1-2kb_low	903	5908	170	110	89	39	80
2-3kb_high	596	2703	37399	217	443	147	0
2-3kb_low	78	586	7749	116	301	134	215
3-6kb_high	8	13	552	28621	4603	20	388
3-6kb_low	0	6	268	8830	1535	74	418

The suffixes “_high” and “_low” refers to the high- and low-quality sequences produced by the IsoSeq sequencing. The data files have the following accession numbers: 1-2kb_high – ERR2869477; 1-2kb_low - ERR2882521; 2-3kb_high - ERR2869478; 2-3kb_low - ERR2869481; 3-6kb_high - ERR2869483; 3-6kb_low - ERR2869484.

### Illumina small RNA sequencing

Small RNAs (below 200 bp) were isolated from a frozen thallus using the Sigma mirPremier kit (Sigma-Aldrich, MO, US) following the manufacturer’s instructions, but including lysis with MagNA beads as described above. The sample was sent to the NSC for library preparation and sequencing. Sequencing library (up to approx. 40 nt fragment size) was prepared and sequenced with Illumina NextSeq 500 as single-end 75 bp reads ( Table 1).

### Draft genome assembly

The basecalled Nanopore reads were processed with Porechop v0.2.2 using default parameters to remove sequencing adapters. Next, the reads were filtered with Nanofilt v2.0.0 ( De Coster *et al*., 2018) to remove reads shorter than 500 bp and average quality below 9. The filtered reads were further error-corrected with LoRDEC v0.7 ( Salmela & Rivals, 2014) using the Illumina reads. First the Illumina reads were quality assessed by removing sequencing adapters and bases with an average quality below 20 (average score across 4 bases), in addition to leading and trailing bases with a quality below 20. This was done using Trimmomatic v0.36 ( Bolger *et al*., 2014). Then lordec-correct (options -k 21 -s 3) was run with the trimmed Illumina reads to correct the filtered Nanopore reads. Then the corrected reads were run through Canu v1.6 ( Koren *et al*., 2017) for further correction (canu -correct with genome Size set to 300 m) and trimming (canu -trim) before assembly (canu -assemble). The assembly was done with two different corrected error rates, 0.144 and 0.146. The two assemblies were almost identical, but the results from using the corrected error rate of 0.144 were used further because the total size was slightly larger and also had the largest contig. The Canu assembly was then polished using the trimmed Illumina reads (described above) by running three rounds of Pilon v1.22 ( Walker *et al*., 2014). The final genome assembly consisted of 5069 contigs with a total length of 171 183 409 nt. The N50 was 43,856 nt and the longest contig of 5,118,963 nt ( Table 3).

**Table 3.** Statistics of the *Hydrurus foetidus* draft genome assembly.

Number of contigs > 1000 bp	Largest contig	Contig N50	Assembly size	Estimated genome size ^a^	Complete and fragmented BUSCO orthologs ^b^	GC (%)
5069	5118963 bp	43856 bp	171 Mb	299.9 Mb	77.2%	45.4

^a^The genome size estimation was based on k-mer frequencies on the Illumina data.

^b^BUSCO was run against the Eukaryota dataset.

## Data availability

All *Hydrurus foetidus* datasets produced in this study are available, study accession number PRJEB29405: https://identifiers.org/ena.embl/PRJEB29405.
