# Supplementary material for: Draft genome assembly and transcriptome sequencing of the golden algae Hydrurus foetidus (Chrysophyceae)
Source: F1000Res. 2019 Oct 7;8:401. Originally published 2019 Apr 8. [Version 3] doi: 10.12688/f1000research.16734.3 (PMC6784874; doi:10.12688/f1000research.16734.3)
Supplement: Peer review history

# Peer review: Reviewer response for version 3

**DOI:** 10.5256/f1000research.22969.r54753

**Published:** 2019-10-07

**Authors:** Daniela Beisser, Stephan Majda

**Affiliations:** 1 Department of Biodiversity, University of Duisburg-Essen, Essen, Germany

The authors answered most of our comments to our satisfaction. Minor comments regarding the methods describing the BUSCO analysis (which version was used?) and genome size estimation (which tool and parameters e.g. k-mer size were used?) are still missing. Overall, we suggest to accept the manuscript.

We confirm that we have read this submission and believe that we have an appropriate level of expertise to confirm that it is of an acceptable scientific standard.

# Peer review: Reviewer response for version 1

**DOI:** 10.5256/f1000research.18291.r49312

**Published:** 2019-08-12

**Authors:** Blake T. Hovde

**Affiliations:** 1 Bioscience Division, Los Alamos National Laboratory, Los Alamos, NM, USA

Review of Draft Genomes: Draft genome assembly and transcriptome sequencing of the golden algae *Hydrurus foetidus* (Chrysophyceae):

 This data note represents a significant contribution to the algal genomics research community in couple different ways. This project represents the first publicly available draft genome of a chrysophyte (golden algae) to date, making it a critical contribution to the study of this particular clade of algae and will be a much-utilized resource for other chrysophyte genome analyses as they emerge. Because this is the first representative of Chrysophyceae to be sequenced is will be a de facto resource, which leads to the next important contribution of the note – which is the importance of the modern sequencing technologies used in development of this genome and transcriptome assembly. The tools used are likely to be the gold-standard tools of *de novo *genome and transcriptome sequencing and assembly. The use of Pacific Biosciences “Isoseq” long read, full length transcriptome sequencing approach will allow this team to generate high quality gene annotations, a great advantage for a new reference such as this genome. Additionally, the use of long read technologies (either Nanopore or PacBio) will be used to generate higher quality genome assemblies with fewer, longer contigs and scaffolds.

 While broad strokes are painted about the contribution of this draft genome to phylogenetic (or phylogenomic) analysis and the importance of the algae as primary producers it is unclear what additional analyses this team will be doing to utilize this genome assembly. Next steps may be to perform biosynthetic pathway analyses and phylogenetic analysis of the Chysophte class or higher level algal/protist analysis. It would be beneficial if the authors could point to some specific follow-ups.

 I was unable to locate the genome assembly using accession number ERZ780628as listed in Table 1. Though, I was able to find the assembly under NCBI accession number: GCA_900617105.1

I confirm that I have read this submission and believe that I have an appropriate level of expertise to confirm that it is of an acceptable scientific standard.

# Response

**Published:** 2019-08-29

**Authors:** Jon Bråte

**Affiliations:** University of Oslo, Norway

We thank the reviewer for the critical reading. We have updated the accession number to the genome assembly (now GCA_900617105.1) and this should work both on EBI and NCBI.

# Peer review: Reviewer response for version 1

**DOI:** 10.5256/f1000research.18291.r47028

**Published:** 2019-05-09

**Authors:** Daniela Beisser, Stephan Majda

**Affiliations:** 1 Department of Biodiversity, University of Duisburg-Essen, Essen, Germany

Please find below the comments to the article “Draft genome assembly and transcriptome sequencing of the golden algae *Hydrurus foetidus* (Chrysophyceae)” by Jon Bråte, Janina Fuss, Kjetill S. Jakobsen and Dag Klaveness.

 The authors present in the article draft genome data for the chrysophyte *Hydrurus foetidus*. *H. foetidus* was chosen because it is a special chrysophyte which is, in contrast to most other chrysophytes, macroscopic, but cells can slide apart under some growth conditions, benthic and thrives under cold conditions in polar and high alpine regions.

 The genome was sequenced using Illumina and Nanopore sequencing, in addition mRNA reads sequenced with PacBio and small RNA reads sequenced with Illumina are provided. The genome was assembled in a hybrid approach, resulting in a length of 171 Mb and 5,069 contigs with a BUSCO completeness of 77%.

 DNA extraction, sequencing and assembly generation are described in detail, but some information is missing, which is listed below.

 Overall, the work is technically sound and should be indexed after minor corrections.

**Minor comments:**

  

1. Abstract: “We combined this data with, to our knowledge, the first draft genome assembly of a chrysophyte algae”. The data is not combined with the draft genome, maybe it should be “We combined this data to create, to our knowledge, the first draft genome assembly of a chrysophyte algae”. Further, there are two draft genomes listed at JGI for *Ochromonas* and *Paraphysomonas* species ( https://genome.jgi.doe.gov/portal/OchCCMStandDraft_FD/OchCCMStandDraft_FD.info.html, https://genome.jgi.doe.gov/portal/ParimpEvaluation_FD/ParimpEvaluation_FD.info.html). They cannot be accessed without registration, but perhaps they should be cited.
2. Abstract: “...belonging to the phylum Heterokonta.” Recent classification of Eukaryotes (Adl *et al *2005, 2012, 2018) ^1^^,^^2^^,^^3^ place the Chrysophyceae into the Stramenopiles which themselves belong to the SAR supergroup. The current classification should be adapted.
3. Abstract: An assembly of 171 Mb was obtained, is this size expected? Is there other data available which suggests this genome size? On the other hand, the k-mer based genome size estimation suggests a much larger genome.
4. Introduction: “multiapproach assembly”, should better be described as hybrid assembly.
5. Culturing of *H. foetidus*: The introduction mentions plastid reduction and heterotrophy in chrysophyte species. *H. foetidus* in called alga, so I assume it is phototrophic, but it would be good to state that somewhere explicitly. Otherwise one would wonder if the cultures were axenic.
6. Concerning the sequencing: Was there quality control performed by the sequencing center (RIN values etc.) before sequencing? Which library preparation protocols were used for the different sequencing data? Was the RNA rRNA-depleted before sequencing? What was the average sequence quality before and after filtering? An overview table of all the sequencing data including quality and number of reads/yield should be added.
7. Nanopore sequencing of genomic DNA: “Albacore v2.1.10 (Linux, Python 3.5 version)” Please provide a reference for Albacore. The information that it was run under Linux with Python 3.5 is probably not necessary.
8. Next line: Table 1 is not the correct reference for the sentence.
9. Table 2: It is not described in the text or caption what “_high” and “_low” means.
10. Table 3: The genome size estimation based on k-mers and BUSCO are not described in the methods section. Maybe the GC content could also be added to the table.

We confirm that we have read this submission and believe that we have an appropriate level of expertise to confirm that it is of an acceptable scientific standard, however we have significant reservations, as outlined above.

## References

[1] : The new higher level classification of eukaryotes with emphasis on the taxonomy of protists. *J Eukaryot Microbiol*.52(5) : 10.1111/j.1550-7408.2005.00053.x 399-451 10.1111/j.1550-7408.2005.00053.x 16248873

[2] : The revised classification of eukaryotes. *J Eukaryot Microbiol*.2012;59(5) : 10.1111/j.1550-7408.2012.00644.x 429-93 10.1111/j.1550-7408.2012.00644.x 23020233PMC3483872

[3] : Revisions to the Classification, Nomenclature, and Diversity of Eukaryotes. *J Eukaryot Microbiol*.2019;66(1) : 10.1111/jeu.12691 4-119 10.1111/jeu.12691 30257078PMC6492006

# Response

**Published:** 2019-08-29

**Authors:** Jon Bråte

**Affiliations:** University of Oslo, Norway

We thank the reviewer for the critical reading. Here are our responses to the minor comments:

1. We have rephrased the sentence and it no longer states that we present the first chrysophyte draft genome assembly.

2. We have rephrased the first sentence of the abstract. And the first time Heterokonta now is mentioned in the introduction we have also added that Adl et al. uses Stramenopila and added the reference. However, we prefer the name Heterokonta as it has seniority to Stramenopila, and is still used by for instance Cavalier-Smith (e.g. Ruggiero et al. 2015; A Higher Level Classification of All Living Organisms. Plos One: https://www.ncbi.nlm.nih.gov/pmc/articles/PMC4418965/).

3. The k-mer estimation was done on the Illumina data alone. While the assembly was done on the Nanopore reads alone (after error correcting with the Illumina reads). Also, k-mer basted estimations of genome size can be inaccurate, especially since we know nothing about the ploidy level or the amount of genetic variation between chromosome copies. Therefore we don't think that this discrepancy is particularly large or surprising.

4. Corrected.

5. We have added a sentence explicitly mentioning that H. foetidus is photosynthetic to the methods section under the paragraph describing the culture procedure. 

6. The PacBio transcriptome sequencing was performed by selecting for polydenylated transcripts (information now added to the manuscript), and for the smallRNA sequencing the totalRNA was size fractionated to remove ribosomal RNA. Information about concentration, integrity and library preparation protocols have been added for the Illumina DNA sequencing under the "Illumina sequencing of genomic DNA" section. Information about concentration and integrity of the DNA for Nanopore sequencing has been added to the section "Nanopore sequencing of genomic DNA". In our opinion, there is not much added value in showing the average sequence quality before trimming/filtering of the different datasets because the various quality thresholds used for the DNA sequences used in the assembly is described. For the small RNA and the transcriptome data we have not performed any filtering or assembly and the raw data is provided.

7. There are different versions of Albacore for different versions of Python. So, we believe it is more specific to keep this information. Albacore is not published (and it is not developed any more) but we added a reference to Oxford Nanopore who developed the software.

8. Corrected.

9. The information has been added.

10. The information has been added.
